# Association between sleeping hours and cardiometabolic risk factors for metabolic syndrome in a Saudi Arabian population

**DOI:** 10.1136/bmjopen-2015-008590

**Published:** 2015-11-30

**Authors:** Jason Brocato, Fen Wu, Yu Chen, Magdy Shamy, Mansour A Alghamdi, Mamdouh I Khoder, Alser A Alkhatim, Mamdouh H Abdou, Max Costa

**Affiliations:** 1 Department of Environmental Medicine, NYU School of Medicine, New York, New York, USA; 2 Department of Population Health, NYU School of Medicine, New York, New York, USA; 3 Department of Environmental Sciences, Faculty of Meteorology, Environmental and Arid Land Agriculture, King Abdulaziz University, Jeddah, Saudi Arabia; 4 Center of Excellence in Environmental Studies, King Abdulaziz University, Jeddah, Saudi Arabia

**Keywords:** metabolic syndrome, Sleep

## Abstract

**Objectives:**

Epidemiological and molecular studies have shown that sleep duration is associated with metabolic syndrome (MtS), a disease that is on the rise in the Kingdom of Saudi Arabia. We aim to investigate the association between sleep duration and selected cardiometabolic risk factors of MtS in a Saudi Arabian population.

**Setting:**

Secondary care was given to the participants. There were 2 participating centres, shopping malls in North and South Jeddah, Saudi Arabia.

**Participants:**

We recruited 2686 participants over a 1-year study period. Participants were selected based on their willingness. The only criterion for exclusion was living in the area (North or South Jeddah) for less than 15 years.

**Planned and primary outcome measures:**

Participants were measured for blood sugar levels, blood pressure and body mass index. All participants were asked to fill out a questionnaire.

**Results:**

There was a positive association between longer sleep duration and obesity, hypertension and hyperglycaemia. The adjusted ORs for obesity, hypertension and hyperglycaemia were 1.54 (95% CI 1.20 to 1.98), 1.89 (95% CI 1.45 to 2.48) and 1.59 (95% CI 1.19 to 2.13), respectively, in participants sleeping >8 h/night, as compared with those sleeping 7 h. The positive associations between longer sleep duration, defined as sleeping >7 h, and the disease status, did not differ from other risk factors such as physical activity and nutrition.

**Conclusions:**

This is the first epidemiological study reporting on the association between sleep duration and cardiometabolic risk factors of MtS in a Saudi Arabian population. Sleep durations of 8 h or greater were found to be associated with all 3 cardiometabolic risk factors: obesity, hypertension and hyperglycaemia, and this relationship was not confounded by quality of nutrition or physical activity levels.

**Strengths and limitations of this study**

- Participants who sleep longer are at a higher risk for metabolic syndrome.
- This is the first epidemiological study to find an association between sleep and metabolic syndrome in a Saudi Arabian population. Cardiovascular disease and diabetes are on the rise in Saudi Arabia.
- This study includes information regarding participants’ eating habits and physical activity.
- This study contains a large population—2686 participants.
- The main weakness of this study is that information on sleep in self-reported.

## Introduction

Metabolic syndrome (MtS) comprises five cardiometabolic risk factors: high blood pressure, obesity, high blood sugar, high triglycerides and low levels of high-density lipoprotein. Occurrence of any three of these risk factors is termed MtS.^1^ MtS has been associated with type II diabetes,^2^ cardiovascular disease (CVD)^3^ and all-cause mortality.^4^ Several epidemiological studies have reported that participants with coronary heart disease (CHD) who have components of MtS have increased mortality rates than do CHD participants without MtS.^5–7^ Given the impact of MtS on an array of health outcomes, it is important to identify all risk factors that lead to the manifestation of MtS far beyond the usual suspects of poor diet and lack of exercise.

One interesting risk factor for MtS that has been identified recently is sleep duration. The National Sleep Foundation recommends 7–9 h of sleep each night for adults aged 18–64 years. Seetho and Wilding^2^ propose that sleep abnormalities form the link between type II diabetes and MtS. Many epidemiological studies have observed associations between MtS and sleep duration.^8–13^ Some have demonstrated a U-shaped association between sleep duration and components of MtS, where participants with short sleep (usually <6 or 7 h) and long sleep (usually >8 or 9 h) duration have higher prevalence of MtS over participants with normal sleep (usually 7–8 h).^10^ ^14^ However, this observation is not the case for all investigations; some studies found that only either short sleep or long sleep duration was correlated with Mts,^11^ ^15^ and others demonstrated that some components of MtS were associated with short sleep duration while other components were associated with long sleep duration.^8^ ^16^ While these studies point towards the notion that amount of sleep influences the development of MtS, the inconsistent findings among the studies warrant further investigations.

Metabolic disorders have become major health concerns in Saudi Arabia, with high rates of obesity among Saudi Arabian citizens.^17^ ^18^ An investigation in 2000 reported that the prevalence of obesity in Saudi Arabian adults was 83%,^18^ and 35% of deaths in Saudi Arabia were due to CVD, which is closely associated with MtS.^19^ A new study released by the WHO in 2014 reported that 60% of Saudi citizens aged 16 years and older were found to be obese. We previously demonstrated that air pollution may be one of the risk factors for MtS in this unique geographic location, as evidenced by disturbance of the expression of genes involved in MtS in both in vitro^20^ and in vivo.^21^ In the present study, we investigated the relationship between sleep duration and MtS in this population, where epidemiological studies on this topic are lacking and MtS burden is increasing.

## Materials and methods

### Study population

We recruited 2686 participants by interviewing individuals visiting two mega-malls in Jeddah, Saudi Arabia, one located in North Jeddah and the other in South Jeddah. A team was present in each mall every Wednesday and Thursday night for a year, from June 2011 to May 2012. Participants were selected based on their willingness. The only criterion for exclusion was living in the area (North or South Jeddah) for less than 15 years. Before interviewing, one of the researchers explained the purpose of the study and the content of the questionnaire, which did not require the participant to be identified by name. Therefore, confidentiality was strictly maintained in all the completed questionnaires. Informed verbal consent, as well as written consent, was obtained from each participant. Participants were informed about the purpose and procedures of the study, and informed that the results would be used for research purposes only. The ethics committee approved the consent procedure.

The questionnaire included age, type of residence, type of work, education, smoking habits, sleeping hours per night, physical activity, hours since the last meal, history of diabetes, hypertension and any other chronic diseases, and weekly frequency of common food intake. After the interview, the participant’s weight and height were recorded wearing light clothes and no shoes. Body mass index (BMI) was calculated according to the formula: BMI=weight (kg)/height (m)^2^.

An accurate blood pressure measurement was carried out. Each participant was asked to sit comfortably for 5 min prior to the examination, during which his/her back was fully supported. Three measurements were taken at 5 min intervals and the mean was recorded. Participants reporting use of antihypertensive drugs were considered hypertensive regardless of their blood pressure reading. Random capillary blood sugar levels were measured by finger pricks. Participants who reported the use of prescribed medications to control diabetes were considered as having diabetes.

### Definitions of parameters

Obesity was defined as BMI>30 kg/m^2^ for men as well as for women. Hypertension was defined as a diagnosis of hypertension, or systolic blood pressure (SBP) ≥140 mm Hg or diastolic blood pressure (DBP) ≥90 mm Hg. Hyperglycaemia was defined as a diagnosis of diabetes, or blood sugar ≥110 mg/dL if it had been ≥8 h after their last meal or blood sugar ≥140 mg/dL if it had been <8 h after their last meal.^8^ ^15^ ^16^ ^22^ ^23^ The short sleeping duration group was defined as individuals sleeping <7 h each night. The long sleeping group included individuals sleeping >7 h and this group was further divided into two subcategories, 8 h and >8 h. Participants who reported sleeping 7 h each night were used as a reference.

Participants were asked how often they ate certain foods each week by giving a value of 0–3 representing eating frequency of <1, 1–3, 4–6 and ≥7 t/week, for each item. For calculating healthy eating scores, food items were first grouped into healthy foods, which include green leafy vegetables, fresh fruits, fish and green tea, and unhealthy foods, which include red meat, processed meat, ice cream, pizza, croissant, French fries, canned meat, carbonated non-diet soft drinks, biscuits, cakes and deep fried foods. The eating frequency of healthy foods was then reversely coded as 3–0 and healthy eating scores were calculated by the sum of the values corresponding to the weekly eating frequencies of each unhealthy or healthy food for each participant. The higher the score, the unhealthier the participant's diet. Participants were asked if they participated in various physical exercises including walking, running, swimming and biking. Participants answering ‘yes’ to any exercise were considered physically active, while participants selecting ‘no activity’ were considered physically inactive.

### Statistical analyses

We first conducted χ^2^ tests to compare differences in distribution of selected population characteristics according to categories of reported sleep duration (<7, 7 and >7 h). We then estimated mean levels of MtS components including BMI (kg/m^2^), SBP (mm Hg), DBP (mm Hg) and blood sugar (mg/dL), by the selected population characteristics. Linear regression was conducted to assess differences in the mean levels of these components by categories of demographics, lifestyle factors and sleep duration.

We used unconditional logistic regression to estimate the ORs for selected cardiometabolic risk factors, including obesity, hypertension and hyperglycaemia, in relation to categories of sleep duration with a sleep duration of 7 h as the reference. We adjusted for potential confounding factors including sex, age (years), smoking, education (years), geographic area, type of residence, physical activity and healthy eating scores. For hypertension and hyperglycaemia, we additionally adjusted for BMI in the models, as we were interested in assessing the association between sleep duration and these outcomes beyond what can be explained by BMI. Linear regression was also conducted to assess the associations between sleep duration and MtS components as continuous dependent variables, with the same adjustments as in the logistic regression models.

We also evaluated whether the effect of longer sleep duration (>7 h) on the metabolic abnormalities differs by selected demographics and lifestyle factors including sex, age, physical activity and healthy eating score. Age, physical activity and healthy eating score were dichotomised by the median value in the overall population. We combined those with 8 h of sleep duration and those with >8 h, because these two groups were positively associated with the outcomes, compared to those with 7 h of sleep duration. Interaction was tested using the cross-product term between the dummy variable for longer sleep duration and the potential effect modifiers, and p values associated with the cross-product terms were used to judge significance of the interaction (p<0.05). All analyses were completed using SAS (V.9.3; SAS Institute Inc, Cary, North Carolina, USA).

## Results

Distributions of selected population characteristics by categories of reported sleep duration are shown in table 1. The proportions of women, those who reside in North Jeddah and live in an apartment, and those who are physically inactive, were higher among participants who reported sleeping more than 7 h per night. Conversely, the proportion of participants who were educated at graduate level was higher among those who reported shorter sleeping hours. There was no clear relationship between age and smoking status with sleep duration.

**Table 1:** Distribution of selected population characteristics by reported sleep duration*

	Overall (n=2686)	Reported sleep duration, hours/night			p Value†
		<7 (n=292)	7 (n=448)	>7 (n=1946)	
Sex					–
Men	1342 (50.0)	148 (50.7)	255 (56.9)	939 (48.3)	0.04
Women	1344 (50.0)	144 (49.3)	193 (43.1)	1007 (51.8)	
Age, years					
≤24	849 (31.6)	100 (34.4)	126 (28.1)	623 (32.0)	0.10
25–34	925 (34.5)	90 (30.9)	141 (31.5)	694 (35.7)	
35–44	525 (19.6)	54 (18.6)	101 (22.5)	370 (19.0)	
≥45	385 (14.3)	47 (16.2)	80 (17.9)	258 (13.3)	
Smoking					
Non-smoker	1941 (72.3)	213 (73.0)	317 (70.8)	1411 (72.5)	0.86
Smoker	745 (27.7)	79 (27.1)	131 (29.2)	535 (27.5)	
Geographic area					
South Jeddah	1327 (49.4)	158 (54.1)	240 (53.6)	929 (47.7)	<0.01
North Jeddah	1359 (50.6)	134 (45.9)	208 (46.4)	1017 (52.3)	
Educational level					
Undergraduate	1280 (47.7)	124 (42.5)	194 (43.4)	962 (49.5)	<0.01
Graduate	1402 (52.3)	168 (57.5)	253 (56.6)	981 (50.5)	
Type of residence					
Popular house	944 (35.2)	116 (39.9)	201 (44.9)	627 (32.2)	<0.01
Apartment	1585 (59.1)	152 (52.2)	223 (50.0)	1210 (62.2)	
Villa	155 (5.8)	23 (7.9)	24 (5.4)	108 (5.6)	
Physical activity					
No activity	1732 (64.5)	167 (57.2)	267 (59.6)	1298 (66.7)	<0.01
Walking/running/swimming/biking	953 (35.5)	125 (42.8)	181 (40.4)	647 (33.3)	

*Data were missing on age for two participants, on educational attainment for four participants, on type of residence for two participants and on physical activity for one participant. Data were also missing on systolic blood pressure for three participants, on diastolic blood pressure for three participants and on capillary blood sugar for one participant. Participants with missing data on any of these variables were excluded from the analyses.

†p Values χ^2^ test or analysis of variance.

Table 2 shows the associations between the selected population characteristics and MtS components including BMI, SBP, DBP and blood sugar. There were equal number of men and women in the study and they did not differ on any of the MtS components except a higher average SBP in men. As age increased, there was a significant increase in all four MtS components. Smokers, participants who were less educated, or those who were physically inactive, had higher levels of all four components. There was no consistent relationship between geographic location (North vs South Jeddah) and type of residence, with the MtS components. Participants who slept longer displayed significantly higher averages for BMI, SBP and DBP, but not for blood sugar.

**Table 2:** Metabolic syndrome components by selected population characteristics

	BMI, kg/m^2^		SBP, mm Hg		DBP, mm Hg		Blood sugar, mg/dL	
	Mean (SD)	p Value*	Mean (SD)	p Value*	Mean (SD)	p Value*	Mean (SD)	p Value*
Sex								
Men	28.4 (5.7)	0.33	129.8 (19.0)	<0.01	79.3 (11.5)	0.42	130.2 (43.9)	0.61
Women	28.1 (5.8)		126.9 (16.6)		79.6 (11.8)		129.3 (46.8)	
Age, years								
≤24	25.4 (5.2)	<0.01	123.6 (12.6)	<0.01	76.8 (10.0)	<0.01	116.4 (28.1)	<0.01
25–34	28.4 (5.4)		125.2 (15.7)		78.8 (11.2)		123.2 (33.6)	
35–44	30.2 (5.2)		132.6 (19.3)		81.5 (12.6)		132.7 (38.3)	
≥45	31.5 (5.3)		140.4 (23.1)		84.2 (12.9)		170.7 (75.7)	
Smoking								
Non-smoker	28.0 (5.7)	0.01	127.5 (17.3)	<0.01	79.0 (11.5)	<0.01	129.4 (47.4)	0.52
Smoker	28.9 (5.8)		130.5 (19.1)		80.5 (12.0)		130.6 (39.6)	
Geographic area								
South Jeddah	27.5 (5.9)	<0.01	128.8 (16.0)	0.20	80.5 (10.1)	<0.01	128.0 (49.4)	0.05
North Jeddah	29.0 (5.5)		127.9 (19.5)		78.4 (12.9)		131.4 (41.0)	
Educational level								
Undergraduate	28.5 (6.0)	0.05	130.8 (19.0)	<0.01	80.9 (12.2)	<0.01	136.1 (52.8)	<0.01
Graduate	28.0 (5.5)		126.1 (16.5)		78.1 (11.0)		123.9 (36.5)	
Type of residence								
Popular house	28.5 (6.0)	0.18	128.5 (18.2)	0.82	78.7 (11.9)	0.04	134.1 (49.3)	0.07
Apartment	28.0 (5.6)		128.1 (17.3)		79.9 (11.3)		126.1 (41.2)	
Villa	28.5 (5.3)		130.0 (21.3)		79.4 (13.7)		140.2 (56.5)	
Physical activity								
No activity	28.6 (5.7)	<0.01	129.4 (19.2)	<0.01	80.5 (12.1)	<0.01	134.1 (48.6)	<0.01
Walking/running/swimming/biking	27.7 (5.8)		126.4 (15.0)		77.5 (10.5)		121.7 (37.6)	
Sleep duration, h/night								
<7	27.6 (5.6)	<0.01	126.1 (15.0)	0.01	76.9 (10.7)	<0.01	127.0 (43.1)	0.48
7	27.8 (5.7)		127.8 (17.6)		78.0 (11.3)		130.8 (47.3)	
>7	28.5 (5.8)		128.8 (18.3)		80.2 (11.8)		129.9 (45.3)	

*p Values from linear regression models.

BMI, body mass index; DBP, diastolic blood pressure; SBP, systolic blood pressure.

There was a positive association between longer sleep duration (>7 h) and obesity, hypertension and hyperglycaemia (table 3). Compared with participants sleeping 7 h, the OR for obesity, hypertension and hyperglycaemia was 1.38 (95% CI 1.05 to 1.79), 1.28 (95% CI 0.97 to 1.71) and 1.17 (95% CI 0.86 to 1.59), respectively, in participants sleeping 8 h, after adjustment for potential confounding factors. The positive association further increased in participants sleeping >8 h, with an OR of 1.54 (95% CI 1.20 to 1.98), 1.89 (95% CI 1.45 to 2.48) and 1.59 (95% CI 1.19 to 2.13) for obesity, hypertension and hyperglycaemia, respectively.

**Table 3:** Associations between sleep duration obesity, hypertension and hyperglycaemia

Reported sleep duration, hours/night	Obesity*		Hypertension†		Hyperglycaemia‡	
	Cases/non-cases, n	OR (95% CI)§	Cases/non-cases, n	OR (95% CI)§	Cases/non-cases, n	OR (95% CI)§
<7	93/199	1.09 (0.77 to 1.53)	56/236	0.77 (0.53 to 1.14)	53/239	0.89 (0.59 to 1.35)
7	146/302	Ref	109/339	Ref	95/352	Ref
8	291/478	1.38 (1.05 to 1.79)	225/543	1.28 (0.97 to 1.71)	185/583	1.17 (0.86 to 1.59)
>8	434/743	1.54 (1.20 to 1.98)	394/781	1.89 (1.45 to 2.48)	310/866	1.59 (1.19 to 2.13)

*Obesity was defined as body mass index (BMI) ≥30 kg/m^2^ for men as well as for women.

†Hypertension was defined as a diagnosis of hypertension, or systolic blood pressure ≥140 mm Hg, or diastolic blood pressure ≥90 mm Hg.

‡Hyperglycaemia was defined as a diagnosis of diabetes, or capillary blood sugar ≥110 mg/dL if ≥8 h after the last meal, or capillary blood sugar ≥140 mg/dL if <8 h after the last meal.

§ORs were adjusted for sex, age (years), BMI (except for the analyses with obesity as the dependent variable), smoking, educational attainment, geographic area, type of residence, physical activity and healthy eating scores.

The short sleeping duration group (<7 h) displayed a very slight increase in risk for obesity (OR 1.09; 95% CI 0.77 to 1.53) and an inverse association with hypertension (OR 0.77; 95% CI 0.53 to 1.14) and hyperglycaemia (OR 0.89; 95% CI 0.59 to 1.35) but none of these observations were significant.

Results from linear regression of MtS components as continuous dependent variables were consistent with the findings based on dichotomised components (see online supplementary table S1). For instance, longer sleep duration (>8 h) was associated with a 1.24 kg/m^2^ (95% CI 0.65 to 1.84 kg/m^2^) increase in BMI, a 2.92 mm Hg (95% CI 1.07 to 4.78 mm Hg) increase in SBP, a 2.71 mm Hg (95% CI 1.47 to 3.9578 mm Hg) increase in DBP and a 1.17 mg/dL (95% CI –3.44 to 5.78 mg/dL) increase in blood sugar, respectively.

**Figure 1:**
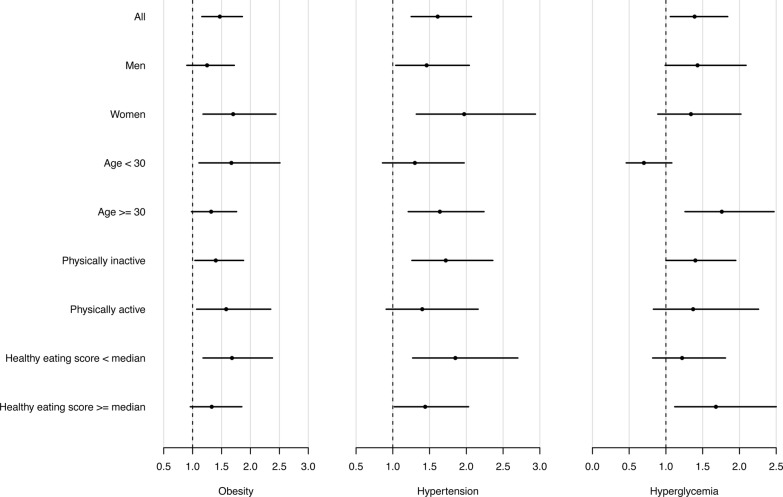
Longer sleep duration and obesity, hypertension and hyperglycaemia in subgroups. ORs were adjusted for sex, age (years), body mass index (BMI; except for the analyses with obesity as the dependent variable), smoking, educational attainment, geographic area, type of residence, physical activity and healthy eating scores. Longer sleep duration was defined as sleep hours per night >7 h; obesity was defined as BMI≥30 kg/m^2^ for men as well as women; hypertension was defined as a diagnosis of hypertension, or systolic blood pressure ≥140 mm Hg, or diastolic blood pressure ≥90 mm Hg; hyperglycaemia was defined as a diagnosis of diabetes, or blood sugar ≥110 mg/dL if ≥8 h after the last meal, or blood sugar ≥140 mg/dL if <8 h after the last meal.

Finally, we explored whether the associations between longer sleep duration (>7 h) and obesity, hypertension and hyperglycaemia differ by selected population characteristics (figure 1). The association between longer sleep duration and obesity was stronger in women, younger individuals and those with a lower than median healthy eating score; however, these interactions did not reach statistical significance (all p for interaction >0.05). Similarly, although there were stronger associations for hypertension among women, older individuals, those who were physically inactive and those with a lower than median healthy eating score, these interactions were not significant (all p for interaction >0.05). There was a significant interaction between longer sleep duration and age in hyperglycaemia (p for interaction <0.01), such that the increased OR (1.76; 95% CI 1.26 to 2.47) was stronger among individuals aged ≥30 years compared with the OR (0.70; 95% CI 0.46 to 1.08) in those aged <30 years (figure 1).

## Discussion

Our data demonstrated that the adverse effect of longer sleep on MtS components starts to display among participants sleeping 8 h and was significantly increased among those sleeping >8 h as compared with those sleeping approximately 7 h. Importantly, the positive association between longer sleep duration and MtS components did not differ by the suspected risk factors for MtS, such as nutrition and physical activity. To the best of our knowledge, this is the first study to demonstrate sleep duration as a potential risk factor for MtS in a Saudi Arabian population, a group from a geographic location not often used in epidemiological studies.

Many epidemiological investigations examining sleep and MtS have generated inconsistent findings. Although a U-shaped association was not evident in our study, other studies^8^ ^16^ ^22^ have reported that short and long sleep durations are associated with MtS components, while some studies only point towards one sleep duration, either short^23^ or long.^15^ While 8 h of sleep is largely viewed as the suggested norm, a large study involving American Cancer Society volunteers concluded that >7 h of sleep is associated with an increased risk of death.^24^ ^25^ These differences may be due to study design, statistical methods, selection bias, or lifestyle differences between populations. Further investigations may reveal that unique genetic and/or social attributes of a Saudi Arabian population allow for shorter sleep periods without negative impacts. However, this hypothesis is not in line with a study by Hall *et al*,^9^ which found, in a mixed population of Caucasians, African Americans and Chinese, sleep correlated with MtS independent of race. Another possibility responsible for the unobserved U-shaped relationship in our study is the large differences in sample size between the <7 h group (N=292) and >7 h group (N=1946). Our study may not have sufficient power in detecting associations for short sleep periods.

Epidemiological studies demonstrated an association between long sleep duration and MtS, as well as molecular studies are uncovering potential mechanisms that may mediate the effects. Several investigations point towards dysfunction in circadian rhythms that occur with altered sleep patterns. The hypothalamus controls a biological clock that is involved in hormone fluctuations and expression of enzymes that regulate metabolism.^26^ Ando *et al*^27^ reported that impairment of peripheral circadian clocks precedes metabolic abnormalities. Leptin deficiency may be the cause of altering the circadian rhythms. While the majority of data on this topic have focused on sleep disruptions that result in shorter sleeping periods, many of the conclusions point towards the notion that any perturbation of the biological clock may impact MtS end points such as obesity and hyperglycaemia. There is evidence suggesting that obesity-associated inflammation may be contributing to longer sleeping times in participants with MtS. Proinflammatory cytokines such as interleukin 1 and tumour necrosis factor-α produced by visceral fat may promote longer sleep and hyperglycaemia as a result of their sleep-inducing^28^ and metabolic^29^ effects. Considering the potential MtS effects of altered circadian rhythms due to longer sleep and how obesity can contribute to longer sleep, this would present a snowball effect for long sleep and MtS. Further studies are needed to confirm which of the two, MtS or sleep alterations, occurs first, but it is likely that there are interindividual differences. Other investigations have reported an increase in depressive symptoms in participants who sleep longer,^14^ ^30^ while a meta-analysis by Pan *et al*^31^ found an association between depression and MtS. Increased inflammation^32^ and activation of the sympathetic nervous system^33^ may mediate these effects.

Some studies^15^ ^30^ that have reported similar findings to our hypothesis demonstrating the association of longer sleep with MtS have suggested that unhealthy diet habits or lack of exercise in long-duration sleepers may be confounding factors; however, these studies did not have data regarding the participants’ dietary habits and involvement in physical activity to test this hypothesis. In this study, we have data on food intake and physical activity. Although not detailed enough to estimate the absolute amount of food intake and levels of physical activity, the data can serve as markers for healthy diet and physical activity. We were able to demonstrate that unhealthy diet habits and lack of physical activity were not confounding factors, as the association between sleep durations and outcomes did not differ by categories of physical activity or healthy eating score.

Our study also has some limitations, although many of these are common in other similar studies. One limitation is that the cross-sectional nature of this study precludes establishing causality of the association. Future prospective studies are needed. The study contained a small number of participants aged 60 years or older (N=40). Sleeping habits of older individuals are likely to vary from those of younger populations so the results may not be generalisable to people aged 60 years or older. Importantly, our data demonstrated that the positive association between longer sleep duration and hyperglycaemia was stronger among individuals aged ≥30 years compared with those aged <30 years. Age-specific association between sleep duration and MtS components thus merits further investigation. Measurement of body fat percentage is a time-consuming procedure and results may vary depending on the person performing the measurements; therefore, BMI was used instead. However, many other studies of MtS evaluating weight have used BMI and there is evidence that BMI may be used instead of body fat percentage when the latter is not available.^34^ The data gathered in our study did not include parameters that would allow for a mechanism to be proposed. Future studies investigating this topic should consider collecting data regarding hormones or specific biomarkers that would allow some insight into the molecular mechanisms mediating the association between long sleep patterns and MtS risk factors. Finally, our analysis regarding the effects of physical activity on long sleep and MtS risk factors had a limitation. Frequency of participation in each physical activity was not considered in the analysis. Given our results demonstrating that physical activity is not a confounding factor in the relationship between long sleep duration and MtS, it is unlikely that frequency of participation would change these outcomes.

Self-reported sleep duration was used in our analyses rather than actual measurement of sleep duration, which is common for many sleep studies. It would be impractical to measure each participant's sleep pattern over an extended period of time in a large population. Responses to self-reported sleep generate some degree of error; however,

previous investigations have found that correlations between self-reported habitual sleep duration with sleep duration measured by polysomnography or actigraphy, have ranged from 0.18 to 0.47,^35^ ^36^ and sleep durations measured by sleep diaries have been reported to be as high as 0.79.^25^

## Conclusion

This is the first epidemiological study reporting on the association of sleep duration with MtS risk factors in a Saudi Arabian population. Sleep durations greater than 7 h were found to be associated with all three risk factors of MtS evaluated: obesity, hypertension and hyperglycaemia, and this relationship was not confounded by quality of nutrition or physical activity levels. Risk factors for MtS are reversible, but if they go unnoticed or untreated, there can be life-threatening consequences; therefore, it is important to identify all elements that influence these risk factors. Further investigations, including molecular studies, are needed to confirm a causative relationship between extended sleep durations and obesity, hypertension and hyperglycaemia. The population examined in this study is from a location rarely reported on in human studies, Saudi Arabia, and offers observations on a behavioural element in relation to MtS in a different geographic and social setting, which is an important first step to understanding the basis of MtS and creating operative therapeutic regimens.
